# Mutations in *NSUN3*, a Mitochondrial Methyl Transferase Gene, Cause Inherited Optic Neuropathy

**DOI:** 10.3390/genes15050530

**Published:** 2024-04-24

**Authors:** Cansu de Muijnck, Jacoline B. ten Brink, Hugoline G. de Haan, Richard J. Rodenburg, Nicole I. Wolf, Arthur A. Bergen, Camiel J. F. Boon, Maria M. van Genderen

**Affiliations:** 1 Department of Ophthalmology, University Medical Center Utrecht, 3584 CX Utrecht, The Netherlands; c.demuijnck@amsterdamumc.nl; 2 Department of Ophthalmology, Amsterdam University Medical Centers, Location AMC, 1105 AZ Amsterdam, The Netherlands; 3 Department of Human Genetics, Section Ophthalmogenetics, Amsterdam University Medical Centers, Location AMC, 1105 AZ Amsterdam, The Netherlands; 4 Department of Human Genetics, Amsterdam University Medical Centers, Location VU, 1081 HV Amsterdam, The Netherlands; 5 Amsterdam Reproduction and Development Research Institute, Amsterdam University Medical Centers, 1105 AZ Amsterdam, The Netherlands; 6 Radboud Center for Mitochondrial Medicine, Departments of Pediatrics and Genetics, Radboud University Medical Center, 6525 GA Nijmegen, The Netherlands; 7 Department of Child Neurology, Emma Children’s Hospital, Amsterdam University Medical Centers, Location VU, 1105 AZ Amsterdam, The Netherlands; 8 Emma Centre for Precision Medicine, Amsterdam University Medical Centers, Location AMC, 1105 AZ Amsterdam, The Netherlands; 9 Department of Ophthalmology, Leiden University Medical Center, 2333 ZA Leiden, The Netherlands; 10 Bartiméus Diagnostic Center for Complex Visual Disorders, 3703 AJ Zeist, The Netherlands

**Keywords:** NSUN3, optic atrophy, inherited optic neuropathy

## Abstract

Inherited optic neuropathies (IONs) are rare genetic diseases characterized by progressive visual loss due the atrophy of optic nerves. The standard diagnostic workup involving next-generation sequencing panels has a diagnostic yield of about forty percent. In the other 60% of the patients with a clinical diagnosis of ION, the underlying genetic variants remain unknown. In this case study, we describe a potentially new disease-associated gene, *NSUN3*, for IONs. The proband was a young woman with consanguineous parents. She presented with bilateral optic atrophy and nystagmus at the age of seven years. Genetic testing revealed the homozygous variant c.349_352dup p.(Ala118Glufs*45) in *NSUN3*, with a segregation in the family compatible with autosomal recessive inheritance. Additional functional analysis showed decreased *NSUN3* mRNA levels, slightly diminished mitochondrial complex IV levels, and decreased cell respiration rates in patient fibroblasts compared to healthy controls. In conclusion, pathogenic variants in *NSUN3* can cause optic neuropathy. Trio whole-exome sequencing should be considered as a diagnostic strategy in ION cases where standard diagnostic analysis does not reveal disease-causing variants.

## 1. Introduction

Inherited optic neuropathies (IONs) are a group of rare genetic disorders characterized by a progressive decline in visual function due to the degeneration of retinal ganglion cells and subsequent optic atrophy. The underlying pathological mechanism predominantly involves impaired mitochondrial function. Notable examples of IONs include Leber’s hereditary optic neuropathy (LHON), dominant optic atrophy, and Wolfram(-like) syndrome. Over the past decade, several novel genes associated with IONs have been identified [1,2,3,4]. Despite advancements in genetic testing, a considerable proportion (40–60%) of patients suspected to have IONs still lack definitive genetic confirmation [5]. Some of these patients may harbor pathogenic variants in genes that have not yet been recognized as being linked to IONs.

IONs can be caused by pathogenic variants occurring in either mitochondrial genes or nuclear genes coding for proteins with a role in energy metabolism. *NSUN3* is a nuclear gene encoding a mitochondrial methyltransferase. NSUN3, or NOP2/Sun RNA Methyltransferase 3, facilitates the methylation of cytosine at the mitochondrial tRNA of methionine, specifically at the wobble position [6,7]. This process is vital for codon recognition. Variants in *NSUN3* have thus far been associated with combined oxidative phosphorylation (OXPHOS) deficiency 48 in two individuals [7,8]. As a nuclear gene with mitochondrial function, *NSUN3* holds promise as a potential candidate gene associated with IONs. However, to date, no (likely) pathogenic variants in this gene have been reported in the literature in association with optic atrophy.

In this study, we described a patient with non-syndromic optic atrophy in the presence of an autosomal recessive disease-associated variant in the *NSUN3* gene. Functional studies confirmed decreased mRNA levels and altered mitochondrial function.

## 2. Materials and Methods

### 2.1. Clinical Examination

Visual acuity was measured using Snellen charts. Visual fields were assessed with Goldmann perimetry. OCT was conducted with Canon Xephilio OCT-A1 (Canon Inc., Kanagawa, Japan). Color vision was assessed with HRR Pseudoisochromatic Plates. Pattern-reversal VEPs were recorded according to the ISCEV (International Society for Clinical Electrophysiology of Vision) standard with the Espion E3 system (Diagnosys LLC, Cambridge, UK) [9].

### 2.2. DNA Sequencing

DNA was extracted from peripheral blood samples according to local protocols. Genetic analysis was performed at the Department of Human Genetics, Amsterdam UMC, the Netherlands. The entire exome was analyzed with next-generation sequence analysis platform HiSeq 4000 (Illumina, San Diego, CA, USA). The intronic regions of the acceptor and donor splice sites up to and including position +/− 6 were analyzed. The reads were mapped against the reference genome (GRCh37/hg19) using BWA mem. Picard tools were used to sort reads and mark PCR and optical duplicates. The GATK Toolbox was used for further quality-control steps and variant calling. Last, SNPEff was used for functional annotation of the variants. Based on the assumed inheritance models, the variants were then assessed for predictive pathogenicity based on various parameters established by standard protocols in line with national and ACMG guidelines [10,11].

Next-generation sequencing panel for inherited optic neuropathies (SeqCap EZ Human Exome Library v3.0, Roche NimbleGen, Pleasanton, CA, USA) included *ACO2*, *AUH*, *C12Orf65*, *CISD2*, *MFN2*, *MTPAP*, *NBAS*, *NDUFS1*, *NR2F1*, *OPA1*, *OPA3*, *RTN4IP1*, *SLC25A46*, *SPG7*, *TIMM8A*, *TMEM126A*, *WFS1*, and *SLC24A1.* Sequencing was performed with HiSeq2500 Illumina (Illumina, San Diego, CA, USA). Alignment and variant filtering were performed according to the same protocols mentioned before.

Mitochondrial mutations m.3460G>A (*MT-ND1*), m.11778G>A (*MT-ND4*), and m.14484T>C (*MT-ND6*) associated with LHON were assessed via Sanger sequencing.

### 2.3. Tissue Sampling and Fibroblast Culture

Punch biopsy specimens were taken from the skin of the affected patient and a healthy unrelated control. Fibroblast cell cultures were grown in M199 medium (Fisher Scientific, Rockingham County, NH, USA) containing 10% fetal calf serum and 1% penicillin–streptomycin. The cells used for the functional studies were from between the seventh and tenth passages.

Extraocular muscle and retina were provided by the Corneabank Beverwijk, Netherlands. The Corneabank obtained permission from the donors for the harvest of the tissues and the use for research purposes in accordance with the international Declaration of Helsinki.

### 2.4. RNA Sampling and Quantitative PCR

Total RNA was extracted from cultured patient dermal fibroblasts and the fibroblasts of the healthy unrelated control with the RNeasy mini kit according to the protocols of the manufacturer (Qiagen, Valencia, CA, USA). Complementary DNA was synthesized from 100 ng of total RNA using oligo-dT primed reactions with Superscript III reverse transcriptase (Thermo Fisher Scientific, Waltham, MA, USA). β-actine primers (sequences on request) were used to confirm the successful synthesis of cDNA. After electrophoresis on 2% agarose gels containing ethidium-bromide, PCR products were imaged using the ChemiDoc MP imaging system (Biorad, Hercules, CA, USA). qPCR analysis was performed using CFX Connect real-time PCR detection system (Biorad). Sequence for forward primer *NSUN3* was 5′-TGGGTCTGTTTGGAATCCTATT-3′ and the reverse primer was 3′-TGCACCACCTTAAATCATTGTTAC-5′ [12]. qPCR reactions were performed in triplicate, and normalization was performed with the 2(-delta delta C(T)) method with GAPDH as reference gene.

### 2.5. Cellular Respiration Studies

Oxygen consumption rates (OCRs) were measured using the Seahorse XFe96 Extracellular Flux analyzer (Agilent, Santa Clara, CA, USA) as previously described by Panneman et al. [13]. Fibroblasts from two different healthy volunteers were used as controls. OXPHOS enzymes were measured and interpreted as previously reported in [13,14].

## 3. Results

The proband was a 14-year-old girl with an uneventful medical history. She had consanguineous parents and a healthy younger brother. She was first examined by an ophthalmologist at the age of 2 due to the suspicion of decreased visual acuity and was diagnosed with myopia and strabismic amblyopia of the left eye. She received spectacles and occlusion therapy for several years without any improvement in visual acuity. When she was 7, she was referred to our tertiary center for further diagnostic evaluation.

**Figure 1:**
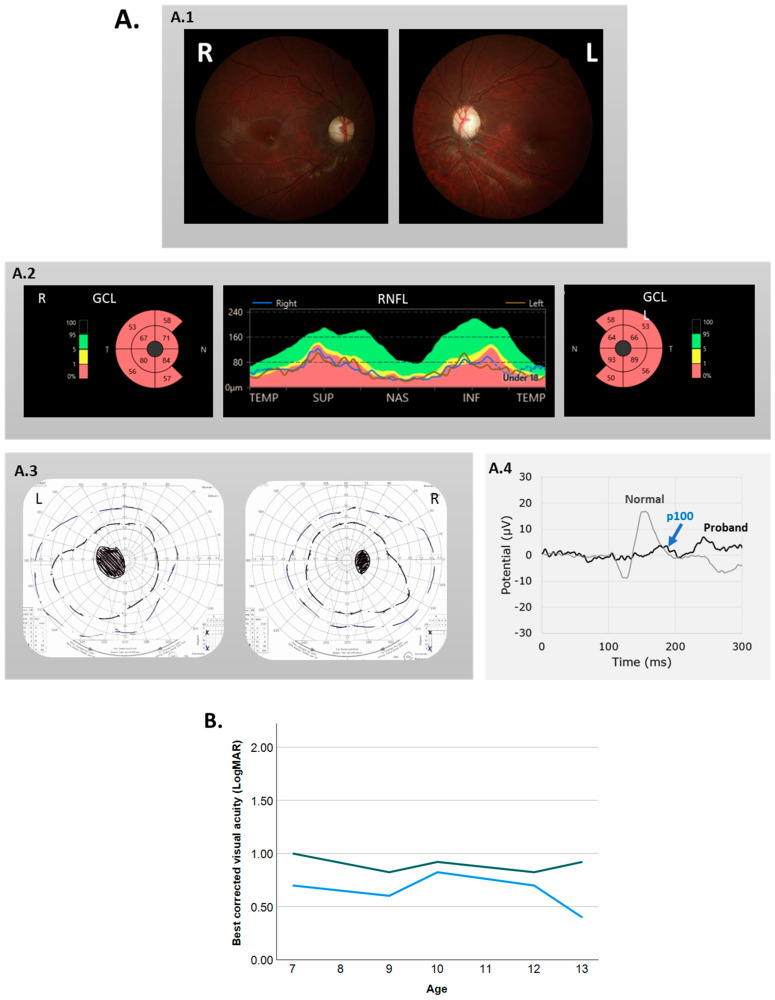
(**A.1**) Color fundus photograph showing bilateral pale optic disks. (**A.2**) The thickness of the ganglion cell layer and the retinal nerve fiber layer were bilaterally decreased on OCT. (**A.3**) Goldman perimetry showing bilateral absolute central scotomas. x marking: size and the intensity of the used stimuli. (**A.4**) Pattern reversal visually evoked potential (VEP) measurements showed a delayed p100 (blue arrow) with decreased amplitude, indicating optic neuropathy. (**B**) Visual acuity remained stable during follow-up period.

At her ophthalmologic examination, the patient had esotropia of the left eye and a rotatory nystagmus in both eyes. Her best corrected visual acuity (BCVA) was 0.68 LogMAR (Snellen 20/100) and 1.0 LogMAR (Snellen 20/200) for the right and left eye, respectively. Visual field testing showed bilateral central scotomas (Figure 1A). An HRR color vision test revealed severe red–green color vision disturbance.

We diagnosed her with bilateral optic atrophy because of pale optic discs on fundoscopy, attenuated ganglion cell and retinal nerve fiber layers on optical coherence tomography (OCT), and small and delayed pattern visual evoked potential (VEP) responses (Figure 1A).

Examination by a pediatric neurologist, including MRI scans of the brain, revealed no neurological explanation for the optic atrophy. The patient had a relatively stable visual acuity during follow-up; the BCVA at her last follow-up at age 13 was 0.39 LogMAR (Snellen 20/50) in the right eye and 0.92 (Snellen 20/200) in the left (Figure 1B).

Trio whole-exome sequencing revealed a novel homozygous loss-of-function variant, c.349_352dup p.(Ala118Glufs*45), in *NSUN3*. Segregation analysis showed that both healthy parents and the healthy brother were heterozygous carriers of this variant, suggesting the autosomal recessive mode of inheritance. Other genetic causes of ION were ruled out with a next-generation sequencing panel (18 genes for ION) and analysis of mitochondrial DNA to exclude LHON.

**Figure 2:**
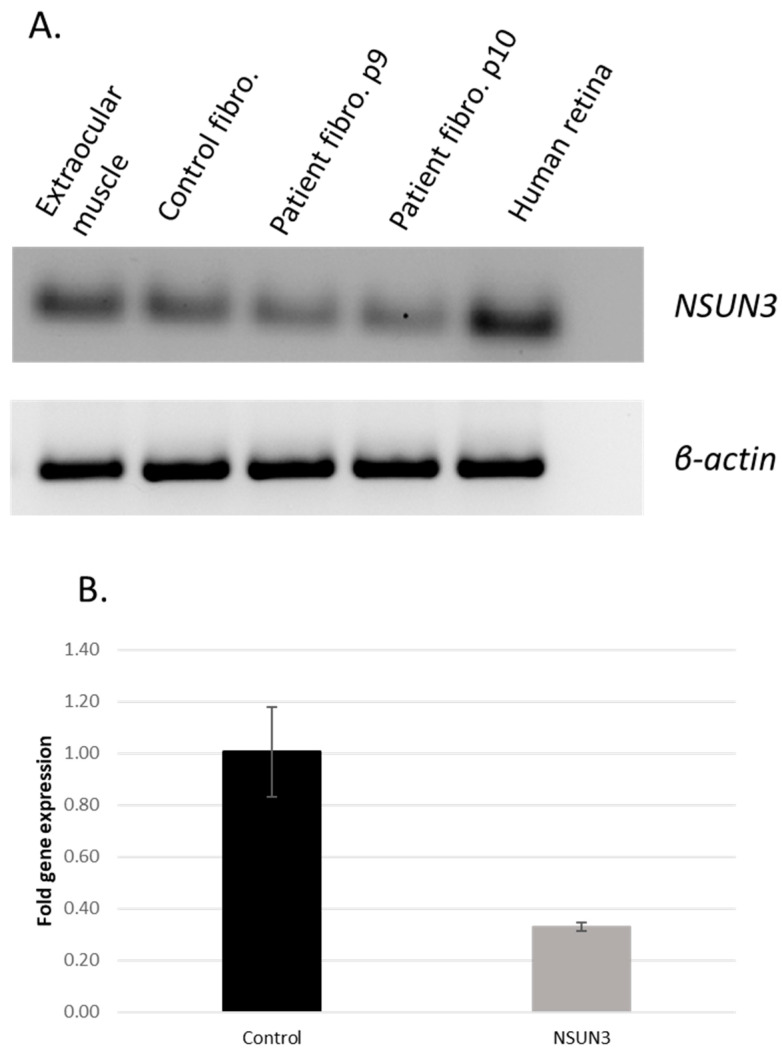
(**A**) Reverse transcription PCR (RT PCR) results for NSUN3 RNA in fibroblasts, extraocular muscle, and human retina. Top, RT–PCR with *NSUN3*-specific primers on various samples. Bottom, RT–PCR with B-actin primers as a control. RT–PCR products of mRNA were obtained from the following human tissues (from left to right): 1. extraocular muscle; 2. fibroblasts of a healthy control; 3. fibroblasts of the patient who carried the *NSUN3* variants, ninth passage; 4. fibroblasts of the patient with the *NSUN3* variants, tenth passage; 5. human retina. (**B**) The results of quantitative PCR showed decreased NSUN3 mRNA in patient fibroblasts compared to healthy controls with GAPDH as housekeeping gene.

We performed additional assays to assess the functional consequences of the aforementioned variant. To investigate the effect of the variant at RNA level, we performed RT–PCR analysis on RNA isolated from patient fibroblasts and compared this with control fibroblasts. We also investigated NSUN3 expression in different control tissues and found variable expression of NSUN3 mRNA in the retina, extraocular muscle, and fibroblasts (Figure 2A). Patient fibroblasts showed the least amount of expression among the analyzed tissues. Quantitative PCR analysis confirmed the significantly decreased amount of NSUN3 mRNA in comparison to the healthy controls (MΔct control = 7.8, SD = 0.24; MΔct patient = 9.5, SD = 0.24; t(4) = −8.1, *p* = 0.001) (Figure 2B).

**Figure 3:**
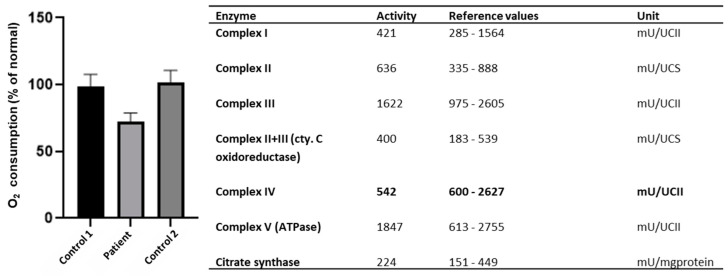
Results of the seahorse respirator, showing decreased oxygen consumption rates in patient fibroblasts compared to two healthy controls (**left**) and slightly decreased levels of complex IV (**right** in bold).

Since earlier studies showed decreased mitochondrial respiration as a result of pathogenic *NSUN3* variants, we explored whether evidence of such decreased activity could also be found in the current patient [7]. Indeed, the measurement of OXPHOS enzyme levels showed slightly decreased levels of complex IV (Figure 3). A cellular respiration assay via seahorse respirator showed a decreased oxygen consumption rate compared to two healthy controls (Figure 3). The oxygen consumption rate through the whole assay can be found in Supplementary Figure S1.

## 4. Discussion

In this study, we described the association of the homozygous variant c.349_352dup p.(Ala118Glufs*45) in the *NSUN3* gene with ION. Functional studies showed decreased respiratory function in patient-derived fibroblasts. Segregation analysis revealed the carriership of the *NSUN3* variant in the healthy parents and brother, in line with autosomal recessive inheritance. Together, these findings support the potential of *NSUN3* as a disease-associated gene for ION.

The function of *NSUN3* was recently uncovered by three different research groups [6,7,10]. In 2016, Van Haute and co-workers demonstrated that low NSUN3 expression leads to mitochondrial translation deficits [7]. They also reported the first patient with combined respiratory chain deficiency due to compound heterozygous loss-of-function *NSUN3* variants. In the same year, Nakano and co-workers showed reduced mitochondrial protein synthesis in *NSUN3* knockout cells [6]. Finally, Haag and co-workers emphasized the role of *NSUN3* in methylation at the wobble position and localized the NSUN3 protein in mitochondria [10]. All three studies described the function of NSUN3 in the methylation of mt-tRNAMet and underlined the importance of this chemical modification for efficient mitochondrial translation.

Currently, two probands are described in the literature with *NSUN3* variants and mitochondrial disease, but optic atrophy has not been described in these cases [7,8]. The first patient had two truncating variants (p.Glu42Valfs*11 and p.Arg99*) in exon 3, and presented with combined developmental disability, microcephaly, external ophthalmoplegia, nystagmus, muscular weakness, failure to thrive, and increased lactate levels. Patient fibroblasts showed decreased NSUN3 mRNA levels compared to healthy controls and patient muscle homogenate displayed combined OXPHOS deficiency [7]. The second patient was an 8-month-old boy born to consanguineous parents, who presented with hypotonia, muscle weakness, lactic acidosis, seizures, and global developmental delay [8]. This patient carried two biallelic, likely pathogenic missense variants in exon 3 of *NSUN3*. Including our study, all reported pathogenic variants have been located in exon 3, which may be a common location for pathogenic variants in *NSUN3*.

The phenotype of our patient with ION differs from the earlier-reported patients, who had a severe phenotype in accordance with global respiratory dysfunction. In contrast, our patient had optic atrophy and nystagmus, and functional studies only showed decreased levels of complex IV. Phenotypic variability is a well-known phenomenon in IONs. In LHON, for example, a small fraction of patients present with severe phenotypes overlapping with other mitochondrial diseases such as MELAS (mitochondrial encephalomyopathy, lactic acidosis, and stroke-like episodes), while most LHON patients develop isolated optic atrophy [13]. This difference in phenotypic severity is attributed to the different variants, differences in the copy number of mitochondrial DNA, and possible gender-specific protective mechanisms [13]. Functional studies in the fibroblasts of the current patient revealed decreased mRNA levels but not a complete depletion. The presence of truncated protein at diminished levels might offer compensation for the deleterious effects within certain tissues, thus potentially accounting for the observed milder phenotype. The exact cause of the phenotypic differences in *NSUN3* mutations remains to be elucidated.

## 5. Conclusions

Specific variants in *NSUN3* are associated with isolated ION. More studies are needed to explore the pathophysiological processes behind disease-causing variants in the *NSUN3* gene. In cases where initial diagnostic approaches do not yield a likely disease-associated gene, trio whole-exome sequencing should be considered to detect rare variants or new disease-associated genes.

## Data Availability

The raw data supporting the conclusions of this article will be made available by the authors on request.

## Supplementary Materials

The following supporting information can be downloaded at: https://www.mdpi.com/article/10.3390/genes15050530/s1, Figure S1: Whole Seahorse assay.
