# Supplementary material for: Mutations in NSUN3, a Mitochondrial Methyl Transferase Gene, Cause Inherited Optic Neuropathy
Source: Genes (Basel). 2024 Apr 24;15(5):530. doi: 10.3390/genes15050530 (PMC11121614; doi:10.3390/genes15050530)
Supplement: Supplementary file 1 [file genes-15-00530-s001.zip › genes-2946037-supplementary.pdf]

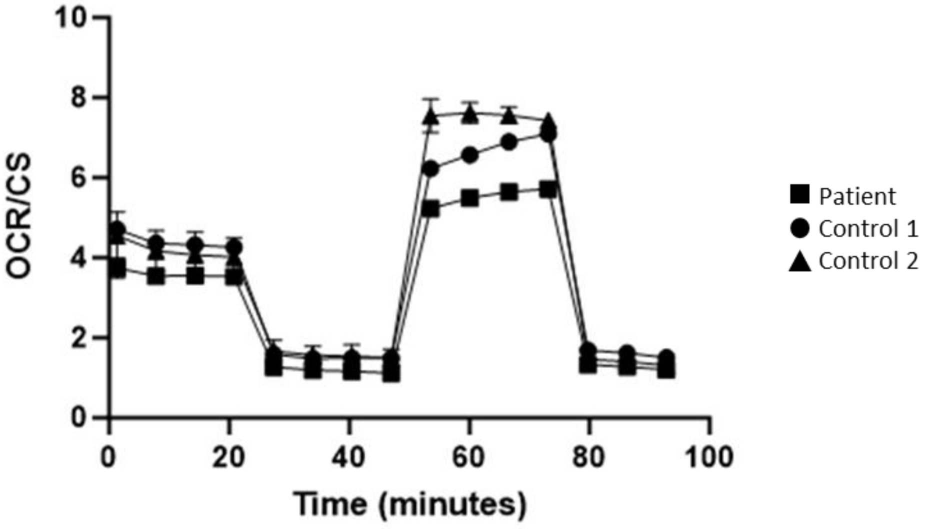

Supplementary Figure S1. Whole Seahorse assay. Results of oxygen consumption rates (OCR) normalized based on citrate synthase activity, shown in a typical Seahorse graph. The results are the averages of 6 wells per cell line. The first 4 datapoints represent the basal oxygen consumption rates. The next 4 datapoints are the OCR in the presence of oligomycin, followed by 4 measurements in the presence of 6 micromolar FCCP resulting in the maximum OCR values shown in Figure 3. The final three datapoints show the OCR in the presence of antimycin A + rotenone.
